# Flexible use of allocentric and egocentric spatial memories activates differential neural networks in mice

**DOI:** 10.1038/s41598-020-68025-y

**Published:** 2020-07-09

**Authors:** Arianna Rinaldi, Elvira De Leonibus, Alessandra Cifra, Giulia Torromino, Elisa Minicocci, Elisa De Sanctis, Rosa María López-Pedrajas, Alberto Oliverio, Andrea Mele

**Affiliations:** 1 Department of Biology and Biotechnology C. Darwin, Sapienza University of Rome, Rome, Italy; 2 Centre for Research in Neurobiology D. Bovet, Sapienza University of Rome, Rome, Italy; 3 Institute of Cellular Biology and Biochemistry, IBBC-CNR, Naples, Italy; 4 Departamento de Ciencias Biomédicas, Universidad Cardenal Herrera-CEU, CEU Universities, Valencia, Spain

**Keywords:** Learning and memory, Spatial memory, Neuroscience, Neural circuits

## Abstract

Goal-directed navigation can be based on world-centered (allocentric) or body-centered (egocentric) representations of the environment, mediated by a wide network of interconnected brain regions, including hippocampus, striatum and prefrontal cortex. The relative contribution of these regions to navigation from novel or familiar routes, that demand a different degree of flexibility in the use of the stored spatial representations, has not been completely explored. To address this issue, we trained mice to find a reward relying on allocentric or egocentric information, in a modified version of the cross-maze task. Then we used Zif268 expression to map brain activation when well-trained mice were required to find the goal from a novel or familiar location. Successful navigation was correlated with the activation of CA1, posterior-dorsomedial striatum, nucleus accumbens core and infralimbic cortex when allocentric-trained mice needed to use a novel route. Allocentric navigation from a familiar route activated dorsomedial striatum, nucleus accumbens, prelimbic and infralimbic cortex. None of the structures analyzed was significantly activated in egocentric-trained mice, irrespective of the starting position. These data suggest that a flexible use of stored allocentric information, that allows goal finding even from a location never explored during training, induces a shift from fronto-striatal to hippocampal circuits.

## Introduction

Goal-directed navigation is one of the most common and conserved cognitive functions, since the ability to go from one place to another in a complex environment is essential for the survival of most animals. Mammals, including humans, can find their way using multiple different navigational strategies, thought to depend on different brain circuits. Traditionally, the hippocampus has been identified as the region mediating world-centered (allocentric) spatial navigation based on the relative position of landmarks in the environment, while the dorsal striatum has been considered the key structure in body-centered (egocentric) navigation, thought to rely on stimulus–response or procedural processing^1–4^. Indeed, lesions of the hippocampus predominantly impair the execution of tasks that require the use of allocentric information, conversely, lesions of the striatum affect tasks dependent on egocentric navigation that requires the association of a particular body turn with the reward^5–9^*.* This view has dominated the field for a long time, but it has been increasingly challenged by accumulating conflicting evidence. It is now well established that a double dissociation exists also within the dorsal striatum, with the dorsolateral (DLS) and the dorsomedial (DMS) compartments mediating respectively procedural versus spatial forms of navigation^10^. Spatial cells have been identified in the DMS^11–13^ and lesions or pharmacological manipulations of the DMS have been shown to have effects resembling hippocampal lesions^14–17^. However, the relative contribution of hippocampus and DMS to allocentric navigation is still a matter of debate. Further, it is clear that navigational abilities do not depend on a single brain region, but rather on a wide network of anatomically interconnected and functionally interacting regions, that extend beyond the hippocampus and the dorsal striatum to include other structures, such as the nucleus accumbens (Nacc) and the medial prefrontal cortex (mPFC)^18–20^.

It has been suggested that the extent to which the hippocampus is involved in spatial navigation could be influenced by the delay between learning and retrieval^21^, the memory load^22^, or the stage of spatial learning^6,23^. A particularly interesting hypothesis posits that the hippocampus could be essential for spatial memory retrieval when a flexible use of spatial representations is necessary to find the goal, as in navigation in a familiar environment through novel routes^24–27^. This view implies that extrahippocampal regions, such as the striatum, would be responsible for guiding navigation through well-acquired spatial routes, however experimental evidence is still limited^24,28^.

Focusing on the flexible use of stored information, we set out to investigate brain activation in CD1 mice trained to find a reward relying on either allocentric or egocentric representations in the cross-maze task, when the mice had to reach the goal using either a novel or familiar route.

The cross maze task has proven to be particularly well-suited for investigating the neural mechanisms of goal-directed navigation. The classic version of this task (also called dual-solution cross maze) takes advantage of the fact that allocentric and egocentric representations of the environment are generally acquired simultaneously, although from different brain structures and on a different timescale^6,29^. Rodents trained to find the reward at a constant position in the T-shaped maze initially use an allocentric strategy, shifting to an egocentric strategy with extended training^6,17,30^. The strategy used by the animal is usually inferred from the behavior in a single probe trial from a novel starting position, never used during training. In order to directly compare the neural circuits implicated in the retrieval of allocentric and egocentric memories we devised two training protocols to induce mice to rely primarily on allocentric or egocentric representations. Mice were trained to obtain the reward either by reaching a particular place relative to extramaze cues (allocentric training) or by making a particular turning response (egocentric training), using two alternative starting points. After eight days of training, mice were tested in a single probe trial either from a novel starting position or from one of the familiar starting positions used during training. This approach has the additional advantage of allowing control over several factors, such as competition between the two strategies at the time of testing, amount of training, or degree of familiarity with the environment, which are known to influence the behavior of rodents in the classic version of the task^6,31^.

To detect neural activation simultaneously in hippocampus, DMS, Nacc and mPFC, we used a non-invasive approach based on immunohistochemical visualization of the immediate early gene (IEG) Zif268, a transcription factor upregulated by memory retrieval^32,33^ and involved in memory reconsolidation^34^. IEGs expression is widely used for defining the neural substrates of behavioral processes, and allows probing of multiple brain regions in intact animals with high anatomical resolution. Finally, we used a pharmacological approach to test predictions based on the results of the brain activity mapping experiments.

## Results

### Differential neural activation after retrieval of an allocentric or egocentric memory from a novel starting position

**Figure 1:**
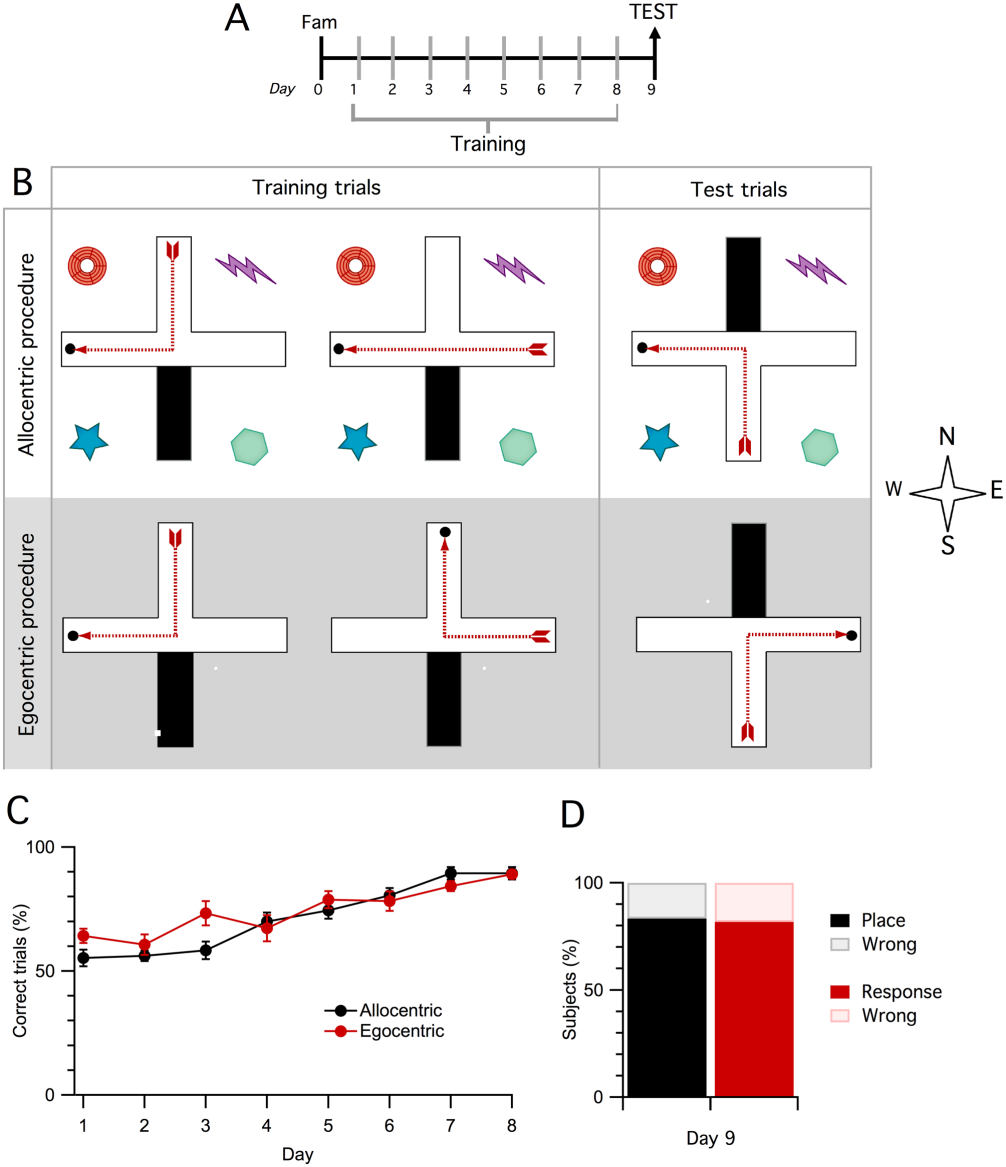
Mice progressively learned both the allocentric and egocentric version of the cross-maze the task. (**A**) Schematic representation of the timeline of the behavioral procedure. After a familiarization session (fam, day 0), mice were trained for eight days (15 trails/day) and received a single test trial on day 9. (**B**) Schematic representation of the apparatus and the training/testing procedure in the allocentric and egocentric versions of the cross maze. (**C**) Mice trained with the allocentric (n = 12) or egocentric (n = 11) procedure improved their performance across days, showing equal acquisition of the task. Symbols represent the percentage of correct trials for each day of training (mean ± s.e.m.). (**D**) In the test trial from the novel starting position on day 9, both allocentric-trained and egocentric-trained mice were able to correctly reach the goal box. Bars represent the percentage of mice that correctly (place or response) or incorrectly (wrong) located the reward at the test trial.

In order to study the neural circuits selectively activated in allocentric-based or egocentric-based goal directed navigation, we developed two different versions of the cross maze task that could be solved using only one of the two strategies (Fig. 1A, B). Two groups of mice were trained for 8 days to find a food reward in the cross maze at a fixed position relative to either extramaze cues or to their body axis. Both groups correctly learned to execute the task and reached high and comparable levels of performance at the end of training, achieving about 90% of correct trials on the last training session, as shown in Fig. 1C (days: F_7,147_ = 31.14, P < 0.0001, repeated measures ANOVA). Overall the two groups performed very similarly, egocentric mice showed a significantly higher number of correct choices, compared to day 1, from day 3 and allocentric mice from day 4 (procedure: F_1,21_ = 1.00, P = 0.328; procedure x days: F_7,147_ = 2.58, P = 0.016, repeated measures ANOVA). There was a significant difference in the latency to reach the goal box between the two groups (Supplementary Fig. S1), with allocentric mice requiring more time than egocentric mice, although both groups significantly decreased the latency across sessions (days: F_7,147_ = 7.85, P < 0.0001; procedure: F_1,21_ = 4.82, P = 0.039; procedure x days: F_7,147_ = 3.14, P = 0.004, repeated measures ANOVA).

During training, entry to one of the four arms of the maze was blocked, so that the animals could be released from only two alternative starting positions. On the test trial, performed on the day after the last training session (day 9, Fig. 1A, B), mice were released from the previously blocked arm, thus in order to reach the reward they had to correctly retrieve information learned during training about the position of the reward, and integrate it with information regarding the current novel starting position. Mice trained with the allocentric procedure had to retrieve information about the position of the reward relative to extra-maze cues and use it to plan a new route in order to reach the goal box. On the other hand, mice trained with the egocentric procedure had to remember the relationship between the start and the goal and continue to use it to turn in the right direction, not taking into account contingent information about the current starting position. As shown in Fig. 1D, the results of the test trial indicate that most of the mice were able to correctly perform the task when placed from a novel starting position (allocentric group: $\chi$^2^ = 5.33, P = 0.02; egocentric group: $\chi$^2^ = 4.45, P = 0.03), and no significant difference was observed between the two groups (Fisher's Exact Test P = 1). This suggests that they were using respectively the acquired allocentric and egocentric strategies to solve the maze.

**Figure 2:**
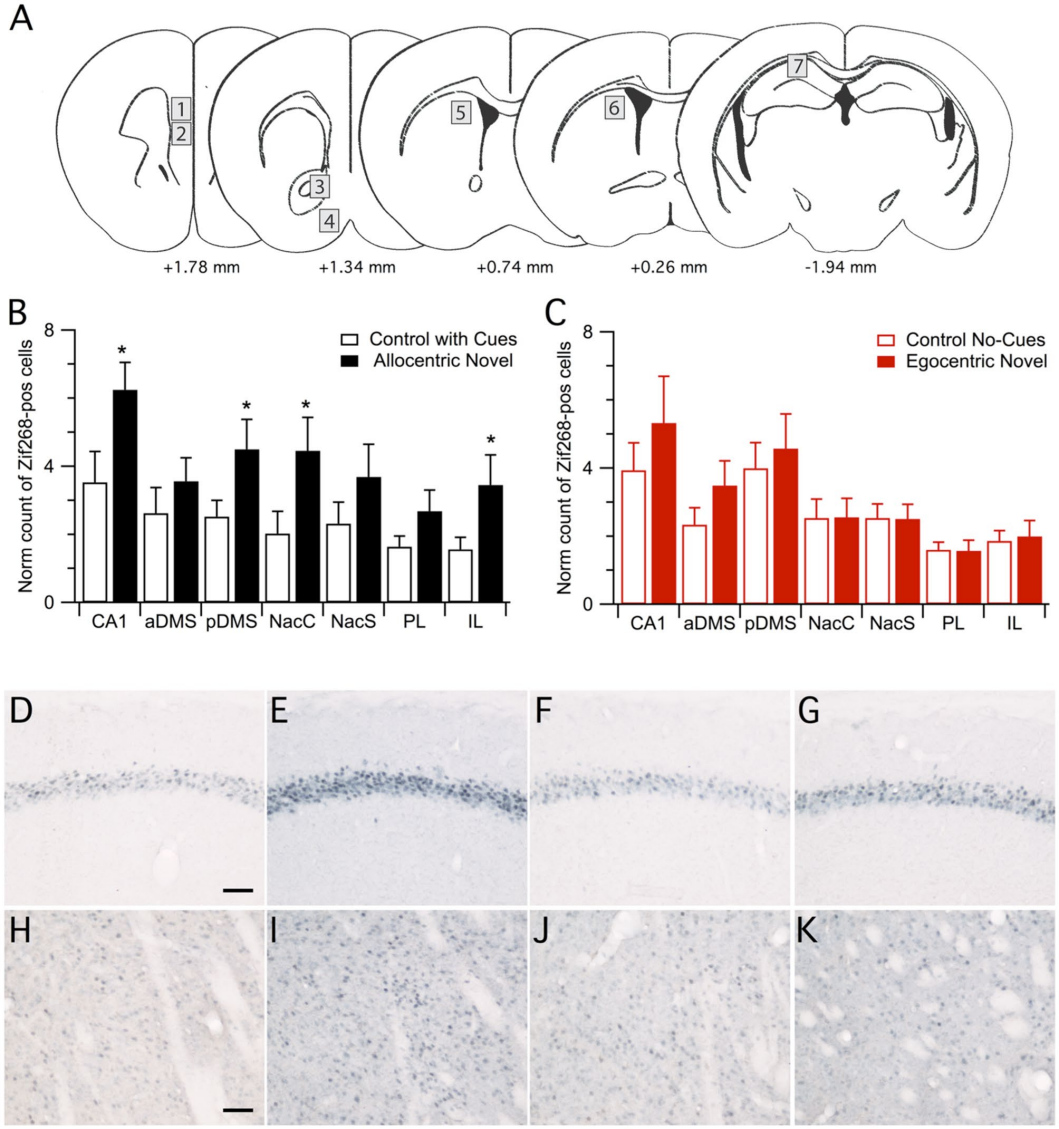
Differential neural activation after retrieval of an allocentric or egocentric memory from a novel starting position. (**A**) Schematic drawings of coronal brain sections showing the sampling areas (grey boxes, 1 = prelimbic prefrontal cortex, 2 = infralimbic prefrontal cortex, 3 = nucleus accumbens core; 4 = nucleus accumbens shell, 5 = anterior dorsomedial striatum, 6 = posterior dorsomedial striatum, 7 = hippocampal CA1). (**B**) Counts of Zif268 stained nuclei from control mice trained in the presence of cues (n = 13) and allocentric-trained mice that performed correctly the final test trial (n = 9). Bars represent mean ± s.e.m. *P < 0.05. (**C**) Counts of Zif268 stained nuclei in sections from control without cues (n = 13) and egocentric-trained mice that correctly performed the final test trial (n = 9). CA1 = CA1 subregion of the hippocampus; aDMS = anterior dorsomedial striatum; pDMS = posterior dorsomedial striatum; NacC = nucleus accumbens core; NacS = nucleus accumbens shell; PL = prelimbic cortex; IL = infralimbic cortex. (**D**–**F**) Representative images of retrieval-induced Zif268 immunoreactivity in hippocampal CA1 (**D**–**G**) and posterior dorsomedial striatum (**H**–**K**) from controls trained in the presence of cues (**D**, **H**) or allocentric-trained mice (**E**, **I**), and from controls trained without cues (**F**, **J**) or egocentric-trained mice (**G**, **K**). Scale bar represents 100 μm.

Brains from mice that achieved a correct outcome in the test trial on day 9 were further processed for quantification of Zif268 expression by immunohistochemistry in the hippocampus, the prefrontal cortex and different components of the striatal complex (Fig. 2A, B). As a control for non-mnemonics factors such as handling, sensory-motor stimulation or stress, we used mice that were trained to retrieve the food at the end of a straight alley and were not required to make any choice (Supplementary Fig. S2). Naive animals were used to normalize Zif268 counts between different experiments. Raw cell counts are shown in Supplementary Table S1. Analysis of the number of Zif268 positive cells showed an overall higher activation in allocentric animals compared to control mice trained in the presence of extramaze cues (training: F_1,20_ = 4.110, P = 0.056; region: F_6,120_ = 9.589, P < 0.0001; training x region: F_6,120_ = 1.460, P = 0.198, repeated-measure ANOVA, Fig. 2B). We found significant differences in CA1 (t_20_ = 2.12, P = 0.046, Fig. 2D, E), posterior dorsomedial striatum (pDMS; t_20_ = 2.14, P = 0.045, Fig. 2H, I), nucleus accumbens core (NacC; t_20_ = 2.16, P = 0.043) and infralimbic prefrontal cortex (IL mPFC; t_20_ = 2.24, P = 0.036) (Fig. 2B). No significant differences were observed for the other regions (t_20_ < 1.64, P > 0.117, Fig. 2B). On the other hand, we did not observe significant differences in Zif268 immunoreactivity between egocentric mice and control animals trained without cues, as shown in Fig. 2C, F, G, J, K (training: F_1,20_ = 0.472, P = 0.499; region: F_6,120_ = 11.265, P < 0.0001; training x region F_6,120_ = 0.763, P = 0.600, repeated measures ANOVA). We did not observe any significant correlation between Zif268 expression and behavioral parameters measured on day 8, such as latency or percentage of correct trials (Supplementary Table S2).

### Differential neural activation after retrieval of an allocentric or egocentric memory from a familiar starting position

**Figure 3:**
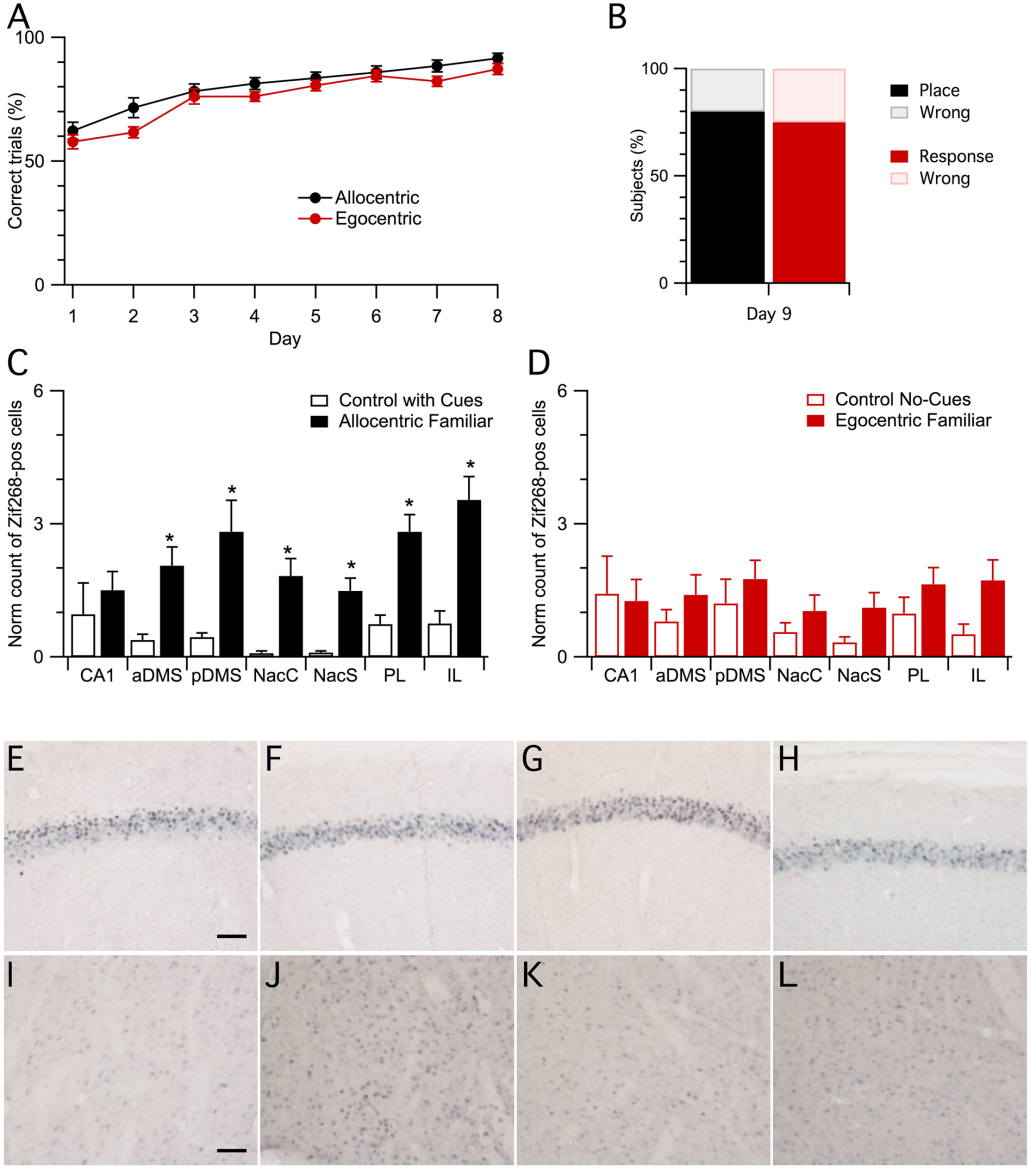
Differential neural activation after retrieval of an allocentric or egocentric memory from a familiar starting position. (**A**) Both allocentric (n = 15) and egocentric-trained mice (n = 12) progressively learned the task during training. Symbols represent the percentage of correct trials for each day of training (mean ± s.e.m.). (**B**) In the test trial from the familiar starting position on day 9, most allocentric-trained and egocentric-trained mice were able to correctly reach the goal box. Bars represent the percentage of mice that correctly (place or response) or incorrectly (wrong) located the reward at the test trial. (**C**) Counts of Zif268 positive cells from control mice trained in the presence of cues (n = 6) and allocentric-trained mice that performed correctly in the final test trial (n = 12). Bars represent mean ± s.e.m. *P < 0.05. (**D**) Counts of Zif268 stained nuclei in sections from control without cues (n = 6) and egocentric trained mice that correctly performed the final test trial (n = 8). Bars represent mean ± s.e.m. CA1 = CA1 subregion of the hippocampus; aDMS = anterior dorsomedial striatum; pDMS = posterior dorsomedial striatum; NacC = nucleus accumbens core; NacS = nucleus accumbens shell; PL = prelimbic cortex; IL = infralimbic cortex. (**E**–**L**) Representative images of retrieval induced Zif268 expression in the CA1 (**E**–**H**) and the posterior dorsamedial striatum (**I**–**L**) from controls trained in the presence of cues (**E**, **I**) or allocentric-trained mice (**F**, **J**), and from controls trained without cues (**G**, **K**) or egocentric-trained mice (**H**, **L**). Scale bar represents 100 μm.

In order to verify to what extent the neural circuits activated in the first experiment were driven by the novel starting position, we trained another cohort of animals as already described, but then we tested them from a familiar starting point. As in the previous experiment, mice progressively acquired the allocentric or the egocentric strategies during the 8 days of training, reaching about 90% of correct responses on the last training session, as shown in Fig. 3A (days: F_7,175_ = 29.19, P < 0.0001, repeated measures ANOVA). The two groups performed similarly, with egocentric mice showing a significantly higher number of correct responses compared to day 1 from day 3 of training and allocentric mice from day 2 (procedure: F_1,25_ = 7.39, P = 0.012; procedure x days: F_7,175_ = 0.59, P = 0.76, repeated measures ANOVA). In both groups we observed a similar significant decrease in the latency to reach the goal box across days (days: F_7,175_ = 7.15, P < 0.0001; procedure: F_1,25_ = 1.17, P = 0.29; procedure x days: F_7,175_ = 0.76, P = 0.619, repeated measures ANOVA; Supplementary Fig. S3).

On the test trial, mice were released from one of the familiar starting positions, thus they were simply required to retrieve and use the strategies learned during training. Both groups showed a similar high level of performance (Fisher's Exact Test, P = 1), as shown in Fig. 3B. Most allocentric trained mice showed a clear ability to find the correct goal box ($\chi$^2^ = 5.4, P = 0.02). Similarly, the majority of egocentric trained mice achieved a correct test trial, although it did not reach significance ($\chi$^2^ = 3, P = 0.08).

We found several regional differences in retrieval-induced Zif268 immunoreactivity between allocentric and control mice, as shown in Fig. 3C (training: F_1,16_ = 9.885, P = 0.006; region: F_6,96_ = 4.843, P = 0.0002; training x region: F_6,96_ = 2.769, P = 0.016, repeated-measure ANOVA). Further analysis showed significant differences between allocentric and control mice in anterior dorsomedial striatum (aDMS; t_16_ = 2.74, P = 0.014), posterior dorsomedial striatum (pDMS; t_16_ = 2.32, P = 0.034, Fig. 3I, J), nucleus accumbens core (NacC; t_16_ = 3.09, P = 0.007), nucleus accumbens shell (NAcS; t_16_ = 3.36, P = 0.004) prelimbic prefrontal cortex (PL mPFC; t_16_ = 3.65, P = 0.002) and infralimbic prefrontal cortex (IL mPFC; t_16_ = 3.57, P = 0.003) but not in CA1 (t_16_ = 0.7, P = 0.5, Fig. 3E, F). No differences were observed in egocentric trained mice compared to controls, as shown in Fig. 3D, G, H, K, L (training: F_1,12_ = 1.498, P = 0.244; region: F_6,72_ = 1.884, P = 0.095; training x region: F_6,72_ = 0.998, P = 0.433, repeated-measures ANOVA). Raw cell counts are shown in Supplementary Table S1.There was no significant correlation between the number of Zif268-positive cells and the behavioral parameters measured on day 8 for allocentric mice (Supplementary Table S2). In the egocentric trained group, we did not observe any significant correlation between Zif268 expression and the mean latency to reach the goal on day 8, however there was a significant negative correlation between the number of Zif268 positive cells and the percentage of correct trials on day 8 for the dorsomedial and ventral striatum (Supplementary Table S3).

### Differential network connectivity after retrieval of allocentric or egocentric memory from a novel or a familiar starting position

In order to analyze the functional connectivity of the network in the different training/retrieval conditions, we computed covariance for pairs of brain regions across subjects, for each trained group^35–37^. First we calculated inter-regional correlations in the "Allocentric Novel", "Allocentric Familiar", "Egocentric Novel" and "Egocentric Familiar" groups, using Zif268-positive nuclei counts, and we generated color-coded matrices to display the correlation coefficient and its significance level, as shown in Supplementary Fig. 4A. We observed a large number of highly significant (P < 0.01) positive correlations in the allocentric-trained groups, and in particular in allocentric animals tested from the novel starting position. We did not find any significant negative correlations. Then, we constructed connectivity network graphs for each group (Supplementary Fig. 4B), where the nodes represent brain regions and the color-coded connections between nodes represent high and significant correlations (R > 0.7, P < 0.05). The graphs clearly show an increase in network density in the "Allocentric Novel" group (19 edges) compared to the others ("Allocentric Familiar", 13 edges; "Egocentric Novel", 12 edges; "Egocentric Familiar", 11 edges. Interestingly, the graphs also reveal that the familiarity of the starting position modulates the functional correlation between the prefrontal cortex and both the hippocampus and the striatum, irrespective of the training procedure. Moreover, in allocentric-trained mice the CA1 showed an increased connectivity with dorsal and ventral striatum subregions, even if it was not significantly activated, as in the Allocentric Familiar group.

### NBQX administration in CA1 before testing selectively impairs the performance of mice tested from a novel starting position, but not from a familiar one, in the allocentric version of the task

With the aim of verifying a causal role of CA1 activation in retrieval of allocentric spatial memory, we inhibited glutamatergic transmission in the hippocampus by injecting the AMPA receptor antagonist NBQX, before testing.

**Figure 4:**
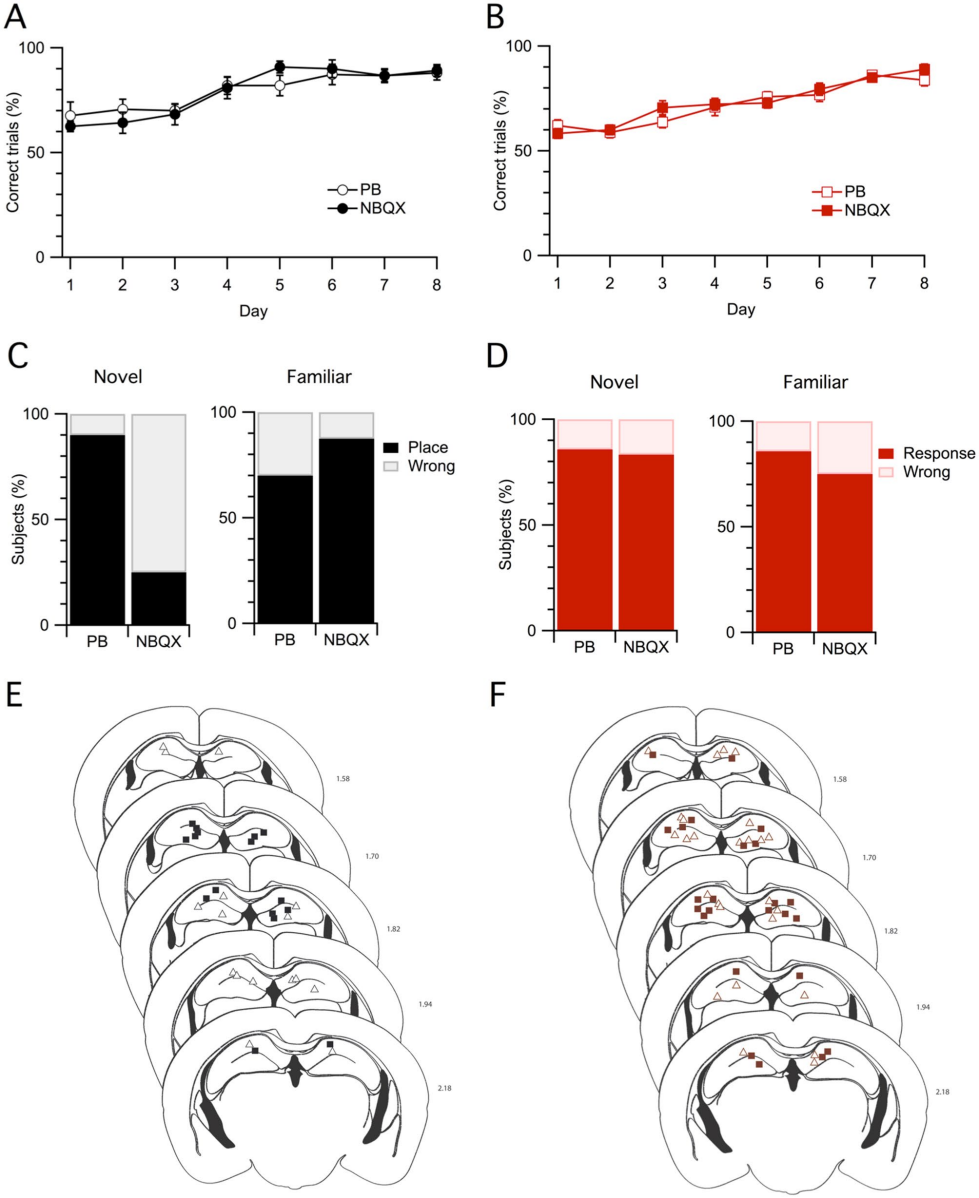
NBQX administration in CA1 before testing selectively impaired the performance of mice tested from the novel starting position in the allocentric version of the task. (**A**) Mice trained with the allocentric procedure, to be injected either with PB or NBQX on day 9, significantly improved their performance across training days and learned the task equally. Symbols represent the percentage of correct trials for each day of training (mean ± s.e.m.). (**B**) Mice trained with the egocentric procedure, to be injected with either PB or NBQX on day 9, significantly increased the number of correct trials across training days and learned the task at a comparable rate. Symbols represent the percentage of correct trials for each day of training (mean ± s.e.m.). (**C**, **D**) NBQX administration in CA1 before testing selectively impaired memory retrieval when mice trained with the allocentric procedure were tested from the novel starting position (**C**), while did not affect mice tested from the familiar starting position (**C**), or trained with the egocentric procedure (**D**). Bars show the percentage of subject that correctly retrieved the food in the maze on the testing trial on day 9. (**E**–**F**) Schematic drawings of coronal brain sections showing the placement of the tip of the injector in the hippocampus for mice trained with the allocentric procedure and injected with either PB (black triangles, N = 10) or NBQX (black squares, N = 8), and mice trained with the egocentric procedure and injected with either PB (red triangles, N = 16) or NBQX (red squares, N = 12).

First, naive mice were implanted bilaterally with cannulas above the hippocampus (Fig. 4E) and then trained in the allocentric version of the cross maze for 8 days. On day 9 mice were randomly allocated to two different groups and were microinjected in the hippocampus with either PB or NBQX, 15 min before the test trial from the novel starting position, which was immediately followed by another training session from the familiar starting positions. The two groups did not differ in the number of correct responses during training (treatment: F_1,16_ = 0.003, P = 0.96, repeated measures ANOVA) nor in the latency to reach the goal (Supplementary Fig. S5A, treatment: F_1,16_ = 0.003, P = 0.96), and both groups significantly improved their performance across days, as shown in Fig. 4A (days: F_7,112_ = 15.59, P < 0.0001; treatment x days: F_7,112_ = 0.84, P = 0.55). Mice that received NBQX before the test trial on day 9, made significantly more errors than the PB-injected controls when tested from the novel starting position (Fig. 4C, Fisher's Exact Test: P = 0.0128). However, when tested from a familiar starting point in an immediately consecutive session, both groups correctly performed the task on the first trial (Fig. 4C, Fisher's Exact Test p = 0.588) and continued to show a percentage of correct trials by the end of the additional training session that was comparable to day 8 (PB day 8: 88 ± 3.41, PB day 9: 88.7 ± 3.45; NBQX day 8: 89.2 ± 2.8, NBQX day 9: 85.8 ± 4.79).

Next, we trained another group of cannula-implanted mice (Fig. 4F) in the egocentric version of the cross maze. On day 9 mice were randomly allocated to either the PB or the NBQX group, and focally injected in the hippocampus with PB or NBQX. As shown in Fig. 4B, PB or NBQX-injected mice did not differ in the percentage of responses during training (treatment: F_1,26_ = 0.27, P = 0.61) and improved their performance across days (days: F_7,182_ = 34.2, P < 0.0001; treatment x days: F_7,182_ = 1.12, P = 0.352, repeated-measures ANOVA). There was no difference in the latency to reach the goal between the two groups (Supplementary Fig. S5B, treatment: F_1,26_ = 0.71, P = 0.41). On the test from the novel starting position, there was no difference between mice administered with PB or NBQX, and the majority of mice in each group were able to correctly reach the goal (Fig. 4D, Fisher's Exact Test: P = 0.673). Both groups continued to perform well on the first trial of training from the familiar starting position (Fig. 4D, Fisher's Exact Test: P = 1), showing a percentage of correct responses in the additional training session on day 9 that was comparable to day 8 (PB day 8: 86.25 ± 1.87, PB day 9: 83.75 ± 2.72, NBQX day 8: 84.99 ± 2.19, NBQX day 9: 88.89 ± 2.51).

## Discussion

Mapping neuronal ensembles by immunohistochemical visualization of the IEG Zif268, we identified a network of anatomically interconnected brain regions, that becomes functional when mice are required to use allocentric information to solve the cross-maze task. We showed that successful memory retrieval in the allocentric version of the cross maze is correlated with the co-activation of a wide network of brain regions, comprising anterior and posterior DMS, NacC, NacS, PL and IL mPFC. Interestingly, the dorsal CA1 of the hippocampus was selectively activated only when allocentric-trained mice were required to reach the goal from a novel starting position. Hippocampal engagement in the test from the novel position, was accompanied by a more limited recruitment of DMS, Nacc and mPFC compared to testing from a familiar starting position. Indeed, only the pDMS, the NacC and the IL mPFC were significantly activated in the test from novel position. These results were confirmed by an inter-regional correlation analysis, which highlighted a prominent functional connectivity of the hippocampus with the other brain structures analyzed in allocentric mice tested from the novel starting point, and a strong reduction in CA1 connectivity with the mPFC in mice tested from a familiar position. These findings support the idea that a flexible use of spatial representations to reach the goal requires the activation of a different network, as compared to navigation through familiar paths.

None of the structures analyzed was significantly activated in egocentric-trained mice, irrespective of the familiarity with the starting position. However, the inter-regional correlation analysis revealed differences in network connectivity between allocentric and egocentric trained mice, most notably a consistent reduction in the correlation of CA1 activity with that of DMS and Nacc.

Pharmacological inhibition of glutamatergic transmission in the dorsal hippocampus, before retrieval, confirmed that this structure was necessary to correctly perform the task only when animals trained to use an allocentric strategy were confronted with a novel starting position, but not when they were released from a familiar one, nor when they were trained with the egocentric procedure.

It is widely accepted that the hippocampus, in particular the CA1 subregion, is essential for allocentric learning. Although supported by a more limited literature, the involvement of the hippocampus in the retrieval of spatial memories has also been suggested^38–40^. For instance, pretest hippocampal inactivation impairs rodent's ability to locate the platform in the water maze^9,41,42^. The results of our pharmacological blockade experiments argue that the differential activation of the hippocampus does not merely reflect detection or processing of novelty^43–46^, or memory updating^47^, but it is instrumental in finding a familiar location when navigating from a novel starting point. These data provide experimental evidence in support of the hypothesis that, in well trained mice, the dorsal hippocampus is essential when stored spatial knowledge has to be flexibly used to find a novel path to a fixed goal, rather than for retrieval per se^24–26^. This is particularly interesting in the context of the recently proposed simulation-selection model that hypothesize that the CA3-CA1 regions of the hippocampus could support flexible navigation by generating and reinforcing novel activity patterns predictive of possible future routes, not yet experienced^48^.

During training in the water maze, subjects are placed in the pool from one of four different positions in each session, and they can explore freely the whole apparatus, thus experiencing multiple routes to the platform location. Typically, in the test trial rodents are released from one of the starting points used during training, or from the center of the pool, that can be considered a novel starting location^9,49^. Based on our results, it would be interesting to compare hippocampal activation in subjects tested from the center or one of the familiar starting points.

Beyond the hippocampus, we analyzed a widely interconnected network comprising the dorsomedial and ventral striatum and the medial prefrontal cortex, based on neuroanatomical and behavioral data supporting a role of these structures in goal-directed navigation. Each of these regions can be further subdivided in at least two compartments based on differences in their afferent/efferent connections^50–54^. Interestingly we found a significant dissociation in Zif268 activation between the different compartments of each region when mice were required to navigate using allocentric information, demonstrating a fine-grained functional distinction within these brain structures dependent on the test demand.

The DMS, and in particular the pDMS, has been previously implicated in forms of hippocampal-dependent spatial navigation^10,11,14–17,55,56^. We observed not only a functional difference between the pDMS and the aDMS, but also not overlapping roles between DMS subregions and the hippocampus. The pDMS was activated when allocentric-trained mice were confronted with either a novel or a familiar starting position. In contrast, the aDMS was significantly activated only in the familiar condition. These results are coherent with previous data in rats trained in the classical version of the cross-maze, showing a prominent role of the pDMS, but not the aDMS, in using a place strategy during the initial stages of learning and when rats where tested from a novel position^17^.

An increasing amount of evidence demonstrates that the ventral part of the striatum, the Nacc, is not only an important component of the reinforcement learning system^57^ but also of the spatial navigation system^49,58–62^. Cells with spatial-related activity have been described in both the NacS and the NacC^58,60^. As in the case of the DMS, we found a dissociation between Nacc subregions. The NacC was activated, compared to controls, both in the novel and familiar conditions, while the NacS was selectively activated when mice were tested from a familiar starting position.

The involvement of the mPFC in spatial navigation is more controversial. The mPFC shows activity correlated to many different behavioral experiences and different stages of memory across a broad range of tasks^40,63–65^. Several studies suggest that the mPFC is important for allocentric navigation^66–74^ as well as egocentric navigation^75^. In the attempt to reconcile these contrasting evidence, Euston and collaborators have recently suggested that the mPFC could be important for flexibly guiding behavior by predicting the most adaptive response based on past experience^64^.

Neurons with spatial correlates, especially related to spatial goals, were found both in PL and IL mPFC^76^ and inactivation of PL-IL caused an impairment in rats trained to shift from a place to a response strategy or *viceversa* in the cross maze, that was interpreted as a deficit in behavioral flexibility involving cross-modal change of strategy^77^. In contrast, PL-IL temporary inactivation did not affect reversal learning, a form of intramodal shift that requires learning to respond to a novel goal location^77^. Our results indicate a differential involvement of mPFC subregions in allocentric navigation, with the IL activated both in the test from the novel and the familiar starting point, and the PL selectively activated in the familiar condition, similarly to the aDMS and the NacS. This result is particularly intriguing in light of increasing evidence showing a dichotomous role of these two mPFC subregions in a variety of processes, including regulation of fear and flexible goal-oriented learning^78–80^.

In the allocentric task we observed a complementary and opposite pattern of activation between the CA1 on the one hand and the PL, NacS and aDMS on the other hand, depending on the familiarity of the starting position. This task-demand dependent shift in the activation from the CA1 to the PL-aDMS-NacS is coherent with the anatomical data, showing that the PL and NacS receive direct and prominent inputs from the hippocampus^52,53^, and that the aDMS is a major target of the PL, compared to the pDMS^50,81^. Further studies will be necessary to elucidate at the circuit level a plausible mechanism for this coordinated shift.

The self-centered egocentric learning is thought to rely primarily on the striatum and the posterior parietal cortex^8,30,82–84^. We did not observe any significant activation of the structures that we investigated, including the DMS and the Nacc. This could mean that these structures, although involved in egocentric learning, are not involved in retrieval of egocentric memories. However, we cannot rule out that in the course of the 8 days of training, in the absence of a confounding alternative strategy, egocentric-trained mice switch to a more habitual responding, which is known to be independent of these structures^30^. Finally, it must be noted that the egocentric procedure requires praxis navigation (i.e. turn right), which is also required in the control condition (i.e. go straight on), although controls, differently from trained mice, do not have alternative routes but are forced to follow a straight path; this might have limited the possibility to detect minor changes in brain activation in egocentric-trained animals.

It is generally believed that allocentric and egocentric information are processed and encoded in parallel^85^ and that rodents acquire simultaneously both types of representations, especially during the initial stages of learning. In this study we set up two procedures to induce mice to use selectively allocentric or egocentric representations in the cross maze. This approach had several advantages compared to the classic version of the task. First, we were able to match the number of trials/training days received by allocentric and egocentric learners, thus avoiding confounding factors that could hinder the interpretation of functional activation experiments, such as differences in habituation to the experimental context and in the level of task performance at the end of training. Moreover, we could identify subjects as allocentric or egocentric learners using both a familiar and a novel starting position in the test trial, and this allowed us to investigate neural activation depending not only on the navigational strategy, but also on the flexible use of the stored representation.

Allocentric-trained mice tested from the novel starting position have to plan a novel route based on their allocentric memory of the goal location, while in the familiar condition they could either rely on the memory of the goal location or retrieve an already experienced and well acquired route. We cannot differentiate between these two scenarios in the case of the familiar condition, but the different patterns of activation observed in the two allocentric testing procedures suggest that animals are relying on different approaches to solve the task in the novel and familiar condition. Even in the familiar testing condition allocentric mice are required to pay attention to environmental landmarks, in order to select the appropriate route depending on the specific starting point, differently from what happens in the egocentric version of the task. The cross maze task is quite simple, involving only one decision point, and mice are confronted with a limited number of possible routes. In the future it will be important to test if accurate retrieval in a more complex environment, where multiple alternative and partially overlapping routes could be learned, would engage differently the allocentric neural network, and especially the hippocampus ^86^. The lack of significant neural activation in egocentric mice suggests that factors common to the two versions of the task, such as motivation, locomotor activity, succesful navigation to the goal, or reward consumption, cannot account for the observed allocentric network activation. More studies will be necessary to dissect the specific role of each subregion, however, looking for a specialized role of each component of the distributed allocentric navigation network could also be misleading, as in a network-based perspective some redundancy would contribute to the preservation of such an important ability, as noted by Ekstrom et al.^20^.

Altogether these data add to increasing evidence showing that allocentric navigation is supported by a distributed neural circuit that goes beyond the hippocampus^28,85,87^ and that retrieval of allocentric and egocentric information is mediated by distinct neural systems in mice^1–4^. More importantly, we dissociated two partially overlapping circuits subserving allocentric navigation, one mediating planning of a new path to a well-known goal location, and the other mediating memory-based retrieval of well learned routes to the goal location, with a significant shift from hippocampus to fronto-striatal circuit modulated by the degree of familiarity of the route.

## Methods

### Subjects

Subjects were CD1 male mice (Charles River, Calco, Italy). Mice were housed in groups of 3–5 in standard breeding cages (26 × 20 × 14 cm) at a constant temperature (22 + 1 °C) and with a 12:12 h light:dark cycle (light on 07.30–19.30). Food and water were available ad libitum unless otherwise stated. At the beginning of behavioral testing mice were 8–10 weeks old and their weight ranged from 34 to 42 gr. All experiments were conducted during the light period.

Every possible effort was made to minimize animal suffering. Procedures were conducted under the authorization N. 169/2009-B from Italian Ministry of Health, according to Italian (DL.116/92) and European laws and regulations on the use of animals in research, and NIH guidelines on animal care.

### Maze

The apparatus consisted of a custom made Plexiglass 4-arms maze and it was elevated 60 cm above the floor in the centre of a dimly illuminated 2 × 2 mt room. The central platform of the maze was 7 × 7 cm. Each arm was 45 cm long and 7 cm wide, and was delimited by 15 cm-high walls. The floor of the maze was black while the walls were transparent. Each arm could be gated either by a clear guillotine-door positioned by the centre of the maze in order to completely block access to the arm, or by a clear guillotine door positioned 15 cm from the end of the arm in order to delimitate an area used alternatively as goal or starting box. A deep food well was located at the end of each arm so that the presence of the food in the well could not be detected visually from a distance. The four arms were conventionally designated as north (N), south (S), est (E), ovest (O) (Fig. 1B).

#### Allocentric and egocentric training procedures

Animals were food-restricted to 80–85% of their body weight 7 days before the beginning of the experiment and maintained in this range throughout. Food pellets consisting of grains of chocolate puffed rice were used as reward in the maze. Mice were habituated to the novel food for 7 days before the beginning of the experiment. Then they were allowed to explore the cross-maze for 10 min and consume 15 pieces of reward scattered throughout the apparatus (day 0, Fig. 1A). Two days after training began. Mice were trained to retrieve the reward with either an allocentric or an egocentric procedure for 8 days (15 trials/day), as described in Fig. 1A, B. Mice were subjected to a single test trial on day 9. During training and testing one arm of the apparatus was blocked by a door positioned by the centre, so that the maze was always in a T-shape configuration (Fig. 1B). In each trial one arm of the T-maze was designated as the goal arm and was baited, one arm was designated as the starting arm and the third was left unbaited, as shown in Fig. 1B. At the beginning of each trial, the mouse was placed in starting box, the door of the box was lifted and the animal was allowed 180 s to retrieve the reward pellet placed in the cup at the end of the goal arm. In each trial mice were allowed to enter only one arm. Mice that correctly reached the reinforced arm were allowed to consume the pellet and then were immediately returned to the starting box. On the contrary when mice reached the non-baited arm they were confined at the end of it for 15 s (correction procedure), before being returned to the starting box. Between trials mice remained undisturbed in the starting box for 40 s. The maze was cleaned with tissue paper between trials and with 50% ethanol between animals. The apparatus was rotated of 90° clockwise everyday in order to discourage the use of potential intra-maze cues. Each mouse was kept in a holding cage for 30 min before training and for 60 min after training every day. An entry was defined as the mouse having at least both forepaws in the last 15 cm of the arm (goal box). Entries into the unbaited arm were scored as errors.

In the *allocentric procedure* the maze was surrounded by several bi- and tridimensional extra-maze cues. The position of the goal arm was maintained fixed relative to the extra-maze cues throughout the experiment and the experimenter stood in a constant position behind the S arm in every trial. On the first trial of the first day of training (t0) the N arm was closed and no reward was placed in the T-maze. The animal was released from the S arm and allowed to reach either the E or the W arm. Independently for each mouse, the non-preferred arm in this trial was blocked during all subsequent training trials. The N arm was designated as the goal arm while the S arm and the E/W arm (depending on t0) were used as starting arms during training, as described in Fig. 1B. The two starting arms were pseudorandomly alternated so that on each daily session the mouse was released an equal number of times from both. On test trials, mice were released from the arm that was blocked during training, while entrance to the opposite arm was prevented (Fig. 1B). This procedure ensured that on the test trial mice were released from a novel starting box, never used during training, and that they were required to choose their non-preferred direction in order reach the goal box. Mice that in the test trial correctly reached the baited N arm, rewarded during training, were perfused one hour after completing the test.

In the *egocentric procedure* the extra-maze cues were removed because in preliminary unpublished experiments we observed that in the presence of cues mice were not able to learn to use the egocentric strategy as well as the allocentric strategy. In the egocentric procedure the relationship between the goal arm and the starting arm was maintained fixed and the experimenter stood behind the starting arm in every trial. On the first trial of the first day of training (t0) the N arm was closed and no reward was placed in the T-maze. Independently for each mouse, the non-preferred turning direction (E or W) was used to determine the relationship between starting arm and goal arm in all subsequent training trials, so that each animal was trained to turn in the non-preferred direction in order to obtain the reward. On test trials, mice were released from the N arm, while entrance to the opposite arm was prevented (Fig. 1B). This procedure ensured that on the test trial mice were released from a novel starting box, never used during training, and that they were required to choose in their non-preferred direction in order reach the goal box. Mice that correctly reached the baited arm in the test trial, thus making the same turning response as during training, were perfused one hour after completing the test.

A different cohort of mice was trained with the allocentric or the egocentric procedure as described above, but on test trials the animals were released from one of the starting box used during training.

Only data from mice that reached a percentage of correct responses $\ge$ 73% on the last two days of training were included in the behavioral analysis. Based on this criterion two animals were excluded from the egocentric group in experiment 2. Only allocentric and egocentric trained mice that made the correct choice on the final test were perfused and their brains used for Zif268 immunostaining experiments. One brain from the allocentric group tested from the novel starting position and one from the egocentric group tested from the familiar position were lost during processing due to problems with perfusion.

#### Control groups

Mice of the control groups were trained and tested with two opposite arms of the maze were blocked so that the apparatus was in a straight alley configuration. Control mice were released from the starting box at one end of the alley and trained to retrieve the food in the cup at the other end, so they never had to choose. All starting arms were alternated during training. The test consisted in a single trial. The allocentric and the egocentric control groups differed only for the presence/absence of extra-maze cues. These groups were added to verify whether differences in Zif268 immunoreactivity were due to non-mnemonic factors such as handling, locomotor activity or sensory stimulation in the apparatus. Naive animals were left undisturbed in the rearing room and then perfused the same day as trained and control animals and they were used as a measure of basal levels of Zif268.

### Immunohistochemistry

One hour after completing the test trial on day 9, each animal was deeply anaesthetized with chloral hydrate (i.p. 500 mg/kg, Fluka, Italia) and transcardially perfused with 50 ml of ice-chilled 0.9% NaCl followed by 100 ml of ice-chilled 4% formaldehyde in PB 0.1 M. Brains were post-fixed for 2 h at 4 °C in 10% formaldehyde in PB 0.1 M and then transferred to 30% sucrose in PB 0.1 M. 30 μm-coronal sections were cut with a freezing microtome (Leica) and stored at − 20 °C in cryoprotectant solution until processing. As described previously^43^, free-floating sections were rinsed several times in 0.1 M PBS (pH 7.4), incubated for 5 min in 3% hydrogen peroxide in PBS and rinsed three times in PBS containing 0.1% Triton X-100 (PBST). After 1 h of incubation in PBST containing 1% BSA and 1% NGS (PBST-BSA-NGS) sections were incubated overnight at room temperature in anti-Zif268 rabbit polyclonal antibody (sc-110; Santa Cruz Biotechnology, USA) diluted 1:5,000 in PBST-BSA-NGS. Sections were washed three times in PBST and incubated for 2 h in biotinylated secondary antibody diluted 1:200 in PBST-1% BSA (goat anti-rabbit IgG; Vector Laboratories, USA). After three washes in PBST sections were incubated for 1 h in avidin-biotinylated peroxidase complex diluted 1:200 in PBST (ABC Kit; Vector Laboratories, USA) and rinsed three times in PBS. Finally, the reaction was visualized using nickel intensified diaminobenzidine (FAST DAB, Sigma, Milan, Italy). The reaction was stopped after exactly 8 min by washing with 0.1 M TBS (pH 7.6). After several rinses in PBS sections were mounted on slides, dehydrated through a graded series of alcohols, cleared and coverslipped. The omission of the primary antibody resulted in no stained nuclei.

### Image analysis

Digital images were acquired at × 20 magnification, using a Nikon microscope equipped with a CCD camera. As previously described^43^, at least three to six non-consecutive sections were stained, digitized and quantified for each brain region and for each subject. Measurements from both hemispheres and from all rostro-caudal levels were averaged. The number of cells showing Zif268 immunoreactivity was measured in the following brain regions: CA1 region of the hippocampus, anterior and posterior medial dorsal striatum, core and shell of the nucleus accumbens, prelimbic and infralimbic prefrontal cortex. Regions were defined according to the mouse brain atlas of Franklin and Paxinos^88^. For each subject brain regions for Zif268 quantification were accurately identified using alternated sections stained with Cresyl Violet.

Quantification of stained cells was carried out using the public domain software Image J (https://rsb.info.nih.gov/ij/). Stained cells were automatically detected based on intensity of staining relative to background and size. These parameters were set based on previous extensive comparisons between automatic and manually generated counts. The experimenter was blind to subject's identity throughout image acquisition and processing.

Sections from groups to be directly compared were processed at the same time and using the same conditions and reagents in order to reduce variability. Only mice that achieved the correct response in the single test trial on day 9 were included in the analysis. The count of Zif268 stained cells from each region was normalized dividing it by the mean count of the naive group for the same region in order to allow comparison of the relative change between the various brain regions and between different experiments.

### Pharmacology

Guide cannulas were implanted 10 days before the beginning of behavioral experiments. Animals were deeply anesthetized with chloral hydrate (i.p., 500 mg/Kg) and placed on a stereotaxic frame (David Kopf Instruments, USA). After carefully drilling two small holes in the skull with a surgical microdrill, a stainless steel guide cannula (length: 7 mm, diameter: 0.5 mm) was lowered 8 mm above the CA1 in each hemisphere of the brain and then fixed to the skull with dental acrylic (Riccardo Ilic, Italy). The following coordinates with lambda and bregma on the same horizontal plane were used: 1.7 mm posterior to bregma, 1.5 mm lateral to midline, 1 mm ventral from the dura, according to the mouse brain atlas (Franklin and Paxinos^88^). Mice were allowed to recover in their home cage for at least 7 days.

Mice were trained with either the allocentric or egocentric procedure for eight days.

Five mice in the allocentric group and five mice in the egocentric group that did not reach at least 73.3% correct responses on the last two days of training were not included in the behavioral analysis.

On day 9, either the AMPA receptor antagonist NBQX (0.0060 μg/μl) or PB (1 M) were administered in the CA1 through an 8 mm-long injection needle connected with polyethylene tubing to a 2 μl Hamilton syringe. A volume of 0.2 μl/side was infused over 2 min using a micropump (Harvard Apparatus, USA). The needle was left in place for additional 2 min to allow diffusion. Mice were allowed to move freely in the cage during the injection. 15 min after the injection mice were subjected to a test trial, and immediately after they were trained for another session from the familiar starting positions.

Upon completion of the behavioral experiment, brains were dissected and immersion-fixed in 4% formaldehyde solution at 4 °C for 3 days. 90 μm-thick coronal sections were then cut with a freezing microtome, mounted onto gelatin-coated slides and stained with 0.5% cresyl violet in order to determine the placement of injections. Only mice with correct placement in the hippocampus were included in the subsequent data analysis.

### Statistical analysis

The number of correct trials and the time to reach the goal box in each trial during training were recorded for each subject and then analyzed by repeated-measures ANOVA, followed by Fisher's LSD post hoc. The percentage of mice that showed correct responses on test trials was analyzed by chi-squared test or Fisher's exact test. Zif268 immunoreactivity data were analyzed by repeated-measures ANOVA. Significant effects were further analyzed by t-test comparison for each brain region. The relationship between Zif268 immunoreactivity and behavioral parameters was analyzed by Pearson's correlation. Level of significance was set at P ≤ 0.05. Data were analyzed with the software SPSS.

### Network correlation and connectivity analysis

Inter-regional Zif268 expression correlation was analyzed by Pearson's correlation. For each trained group, Pearson's correlation was calculated in pairs between all 7 brain regions analyzed. Correlation matrices were generated using Pearson’s R values and the corresponding P values (level of significance was set at P ≤ 0.05), and showed as color coded correlation matrices^36,37^.

Pearson's inter-regional correlation data were used to generate the network graphs, with a threshold of R > 0.7 and P < 0.05. Each node in the graph represents a brain region and the connections between nodes (edges) represent significant and above threshold correlations. The node size is proportional to the normalized count of Zif268 stained nuclei for that brain region. The color of the edge reflects the correlation coefficient and the thickness is proportional to the corresponding P value. The network graphs were generated using Cytoscape^89^.

## Supplementary information

Supplementary information
