# Supplementary material for: Flexible use of allocentric and egocentric spatial memories activates differential neural networks in mice
Source: Sci Rep. 2020 Jul 9;10:11338. doi: 10.1038/s41598-020-68025-y (PMC7347635; doi:10.1038/s41598-020-68025-y)
Supplement: Supplementary file 1 — Supplementary information [file 41598_2020_68025_MOESM1_ESM.pdf]

Flexible use of allocentric and egocentric spatial memories activates differential neural networks in mice.

Arianna Rinaldi[1,2,§,*], Elvira De Leonibus[3,§], Alessandra Cifra[1], Giulia Torromino[1], Elisa Minicocci[1], Elisa De Sanctis[1], Rosa Lopez-Pedrajas[1,4], Alberto Oliverio[1,2] and Andrea Mele[1,2]

1 Department of Biology and Biotechnology C. Darwin, Sapienza University of Rome, Italy;

2 Centre for Research in Neurobiology, Sapienza University of Rome, Italy;

3 Institute of Cellular Biology and Biochemistry, IBBC-CNR, Italy;

4 Departamento de Ciencias Biomédicas, Universidad Cardenal Herrera-CEU, CEU Universities, Spain;

§ These authors contributed equally

*** Corresponding author, email: arianna.rinaldi@uniroma1.it**

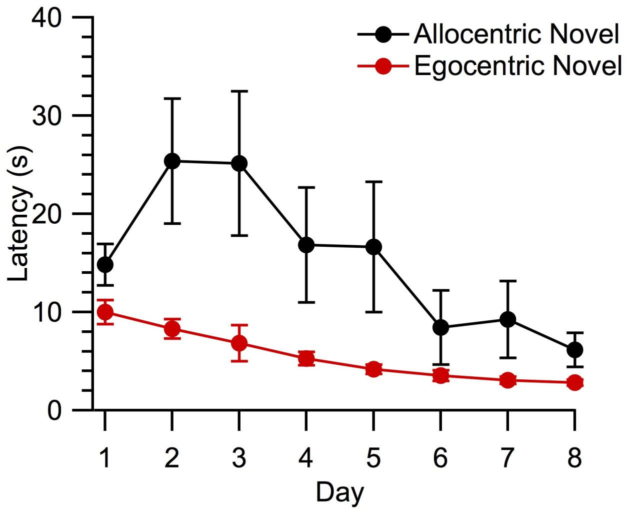

Supplementary Figure S1.

Latency to reach the goal box during training in allocentric and egocentric mice. Mice trained with the allocentric (n=12) or egocentric (n=11) procedure improved their performance across days. Data are presented as mean $\pm$ s.e.m.

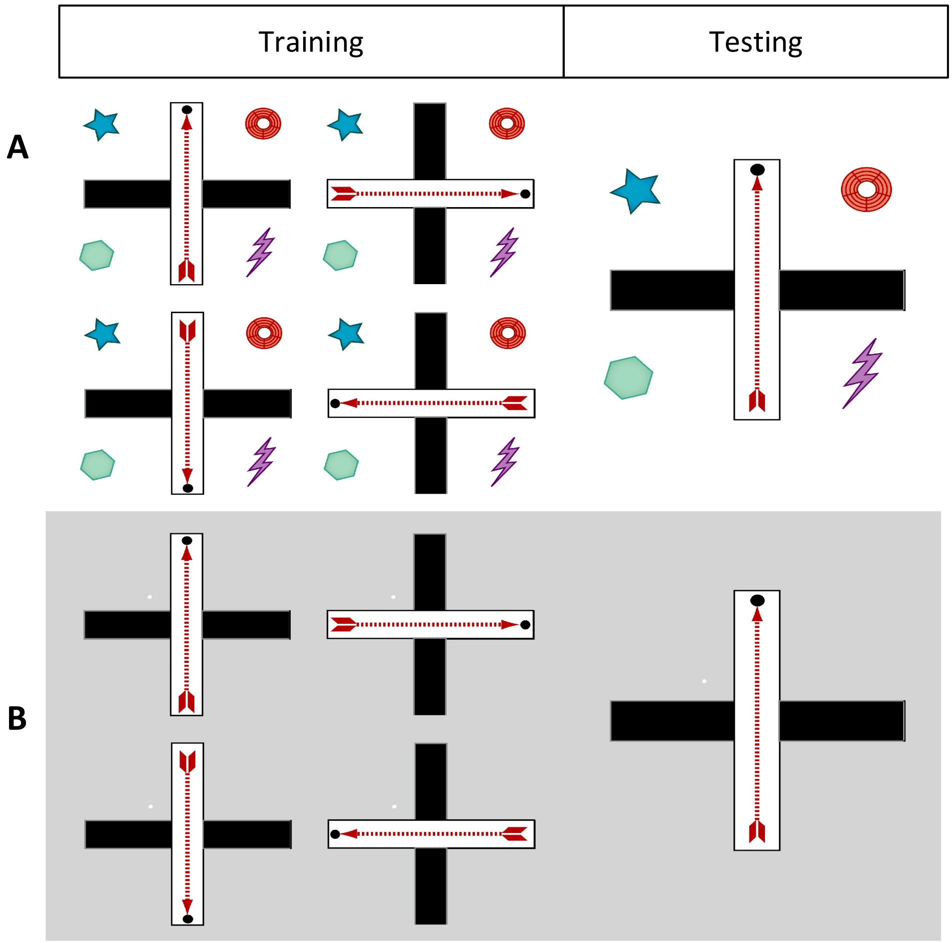

Supplementary Figure S2.

Schematic representation of the apparatus and the training/testing procedure for controls with cues (A) and control without cues (B).

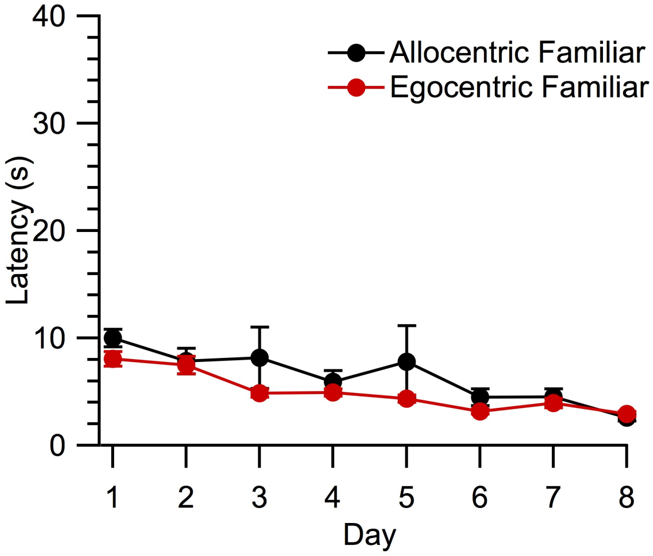

Supplementary Figure S3.

Latency to reach the goal box during training in allocentric and egocentric mice. Mice trained with the allocentric (n=15) or egocentric (n=12) procedure improved their performance across days. Data are presented as mean ± s.e.m.

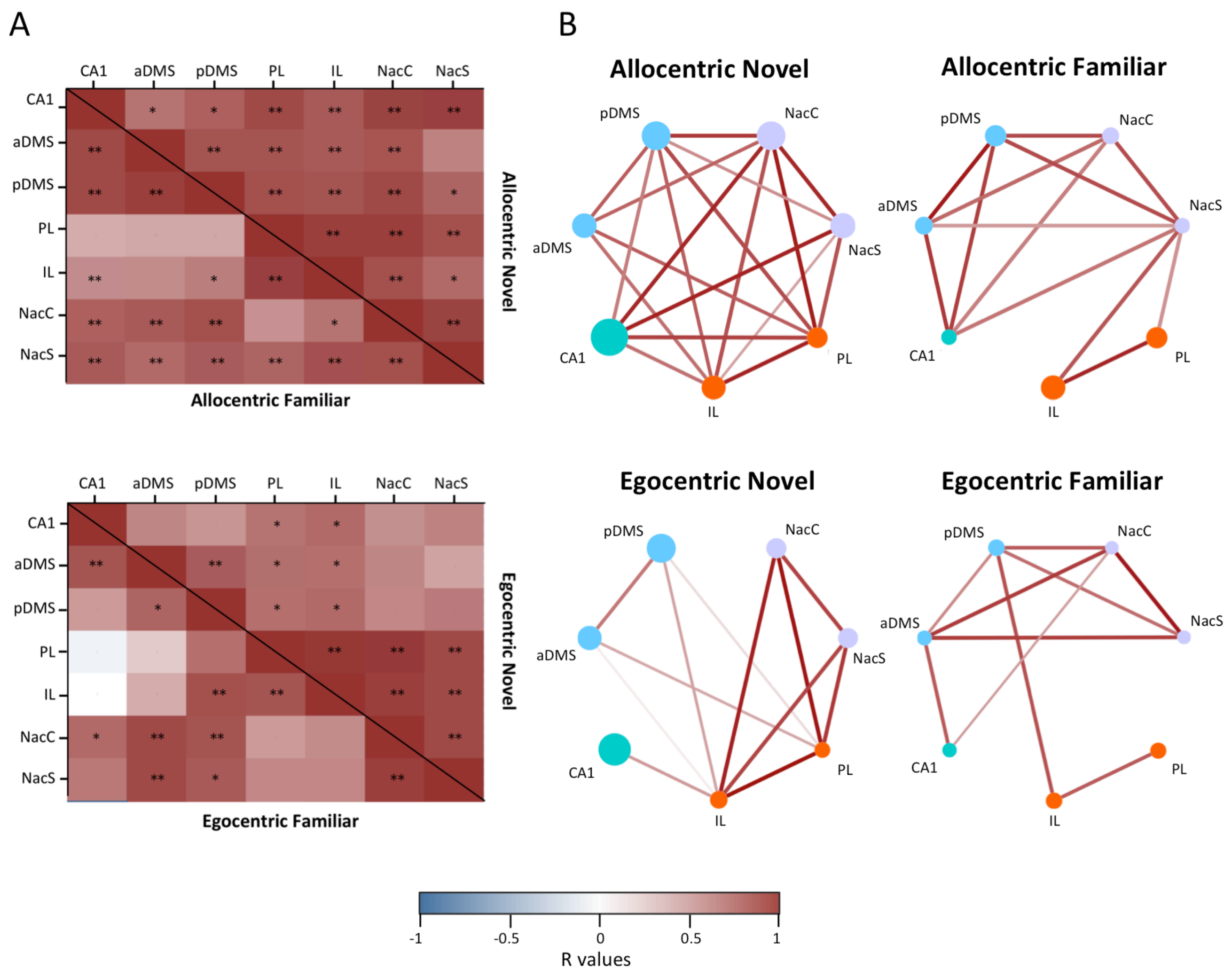

Supplementary Figure S4.

(A) Pearson's correlation color-coded matrices, showing the inter-regional correlation of Zif268 counts for the Allocentric novel, Allocentric familiar, Egocentric novel and Egocentric familiar groups. The color of each cell in the matrix reflects the correlation coefficient, and the asterisk reflects the corresponding P value: *P<0.05; **P<0.01. (B) Network connectivity graph showing significant correlations with strong correlation coefficient (R>0.7; P<0.05). The nodes represent brain regions and the connections between nodes (edges) represent significant correlations. The color of each edge reflects the correlation coefficient, and the tickness of the line is proportional to the corresponding P value. The node size reflects the normalized count of Zif268 stained nuclei for that brain region.

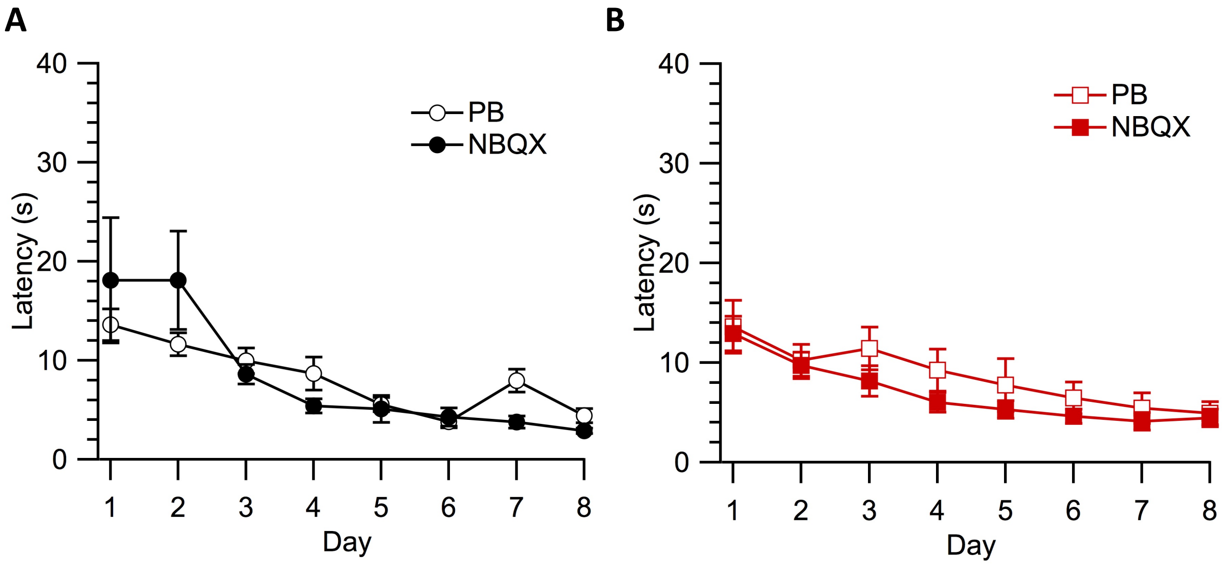

Supplementary Figure S5.

Latency to reach the goal box during training in allocentric-trained (A) and egocentric mice mice(B), to be injected with either PB or NBQX on day 9. Data are presented as mean + s.e.m.

	CA1	aDMS	pDMS	NacC	NacS	PL	IL
Naive	34 ± 15	52 ± 23	27 ± 11	24 ± 8	16 ± 5	36 ± 8	12 ± 5
Allocentric Novel	**213 ± 27**	184 ± 36	**123 ± 24**	**107 ± 24**	61 ± 16	97 ± 23	**41 ± 10**
Control with cues	120 ± 31	136 ± 39	69 ± 13	49 ± 16	38 ± 10	60 ± 11	18 ± 4
Egocentric Novel	181 ± 47	180 ± 37	125 ± 28	62 ± 13	41 ± 7	57 ± 12	24 ± 5
Control no-cues	134 ± 27	121 ± 26	109 ± 21	61 ± 13	42 ± 7	58 ± 8	22 ± 4
Naive	74 ± 14	66 ± 17	35 ± 10	29 ± 7	25 ± 7	28 ± 7	11 ± 4
Allocentric Familiar	112 ± 31	**135 ± 28**	**100 ± 25**	**53 ± 11**	**37 ± 7**	**78 ± 11**	**39 ± 6**
Control with cues	72 ± 52	25 ± 8	16 ± 3	2.4 ± 1.4	2.4 ± 0.8	20 ± 6	8.3 ± 3
Egocentric Familiar	94 ± 36	91 ± 30	62 ± 15	30 ± 10	27 ± 8	45 ± 10	19 ± 5
Control no-cues	106 ± 63	52 ± 17	43 ± 19	16 ± 6	8.1 ± 3	27 ± 10	5.6 ± 2.5

Supplementary Table S1.

The table shows the raw counts of Zif268 stained nuclei for each group as mean ± s.e.m. Sections from the groups above the double line were processed together as one batch (data shown in Figure 2). In the same way, sections from the groups below the double line were processed together (data shown in Figure 3). Significant differences between a trained and the matched control group (line immediately below) were highlighted in bold ($P<0.05$, *t-test*).

	Allocentric Procedure		Egocentric procedure	
	Latency d8	% Correct d8	Latency d8	% Correct d8
CA1	-0.61, P=0.07	-0.08, P=0.84	-0.43, P=0.25	0.29, P=0.44
aDMS	-0.52, P=0.15	-0.41, P=0.27	-0.24, P=0.54	-0.40, P=0.29
pDMS	-0.66, P=0.05	-0.007, P=0.99	-0.06, P=0.87	-0.26, P=0.50
Nacc Core	-0.57, P=0.11	-0.24, P=0.54	-0.05, P=0.89	0.26, P=0.50
Nacc Shell	-0.52, P=0.15	-0.12, P=0.75	0.11, P=0.78	0.47, P=0.20
PL	-0.51, P=0.16	-0.30, P=0.42	-0.06, P=0.88	0.21, P=0.59
IL	-0.57, P=0.11	-0.34, P=0.37	-0.16, P=0.68	0.21, P=0.59

Supplementary Table S2.

The table shows Pearson's correlation value (R), and the corresponding P values, between Zif268 counts after testing from the novel starting position and behavioral parameters measured on day 8, for allocentric (n=9) or egocentric (n=9) mice.

	Allocentric Procedure		Egocentric procedure	
	Latency d8	% Correct d8	Latency d8	% Correct d8
CA1	0.33, P=0.29	-0.28, P=0.37	0.36, P=0.38	-0.47, P=0.24
aDMS	0.47, P=0.12	-0.15, P=0.64	0.51, P=0.2	-0.78, *P=0.02*
pDMS	0.39, P=0.21	-0.11, P=0.72	0.6, P=0.12	-0.78, *P=0.02*
Nacc Core	0.32, P=0.32	-0.07, P=0.83	0.43, P=0.28	-0.76, *P=0.03*
Nacc Shell	0.02, P=0.94	0.11, P=0.73	0.36, P=0.38	-0.78, *P=0.02*
PL	-0.29, P=0.35	0.48, P=0.11	0.11, P=0.8	-0.42, P=0.3
IL	-0.24, P=0.45	0.31, P=0.32	0.47, P=0.24	-0.67, P=0.07

Supplementary Table S3.

The table shows Pearson's correlation value (R), and the corresponding P values, between Zif268 counts after testing from the familiar starting position and behavioral parameters measured on day 8, for allocentric (n=12) or egocentric (n=8) mice.
